# Histologic evaluation of femoral nerve demyelinating and axonal neuropathy in Wistar rats due to alendronate intake: a randomised study

**DOI:** 10.1186/s40709-020-0112-z

**Published:** 2020-02-18

**Authors:** Papamitsou Theodora, Papakoulas Apostolos, Papaliagkas Vasileios, Karachrysafi Sofia, Dietrich Eva-Maria, Sioga Antonia

**Affiliations:** Laboratory of Histology and Embryology, Faculty of Medicine, Aristotle University of Thessaloniki, 54124 Thessaloniki, Greece

**Keywords:** Bisphosphonates, Alendronate, Femoral nerve, G-Ratio

## Abstract

**Background:**

Bisphosphonates (BPs) are forceful inhibitors of osteoclast-mediated bone resorption. Long-term BP use is associated with multiple rare but severe adverse effects. The objective of this study was to investigate the possible effects of BPs in the structure of femoral nerve. Specimens from the femoral nerve of ten female 12-month old Wistar rats were used as control group and ten female 12-month old Wistar rats to which Alendronate (Fosamax, Merck) was administered per os for 13 weeks, were used as research group. Samples were observed under a Transmission Electron Microscope. G ratio measurements and statistical analysis with SPSS program were also performed.

**Results:**

The control group showed no major changes of the nerve’s histologic image, with the exception of some spots of thickness of the nerve myelin sheath. The research group showed major morphological changes which varied from partial disorganization or thickening of the myelin to severe myelin thickening and axon strangulation. A statistically significant difference of the G ratio between the two groups was observed.

**Conclusions:**

The reported values (found in literature) for the morphologic measurements of the femoral nerve in Wistar rats are not complying with the ones we found in our study. There was a significant reduction of all three variables (the mean axon like diameter, the myelin thickness, G ratio) studied in the femoral nerve of the research group in contrast to control group. Our study demonstrates a possible correlation between alendronate administration and femoral nerve’s function, nevertheless due to the small specimen further research is needed.

## Background

Bisphosphonates (BPs) are forceful inhibitors of osteoclast-mediated bone resorption. During the last decades, their role as experimental agents was upgraded to becoming the treatment of choice for a variety of bone disorders, mostly osteoporosis, Paget’s disease and hypercalcaemia of malignancy [1]. Their high affinity for bone mineral, but not other tissues, indicates them as the ideal candidates for treatment of bone diseases [2].

Bisphosphonates are chemically stable derivatives of inorganic pyrophosphate (PPi), a naturally occurring compound in which two phosphate groups are linked by esterification [2]. In BPs, the oxygen bridge has been replaced by a carbon with various side chains (P–C–P) [3]. It is clear that suppressing osteoclast bone resorption is their principal biologic effect, with the potency of this effect dependent on the side chains [1]. They bind to hydroxyapatite crystals, preferentially incorporating into sites of active bone remodeling and reduce bone turnover by inducing osteoclast apoptosis. The first generation of non-nitrogen-containing BPs (etidronate, clodronate, and tiludronate) inhibit osteoclasts by inhibiting the ATP-dependent cellular processes, while the nitrogen-containing BPs (alendronate, risedronate, ibandronate, pamidronate, and zoledronic acid) inhibit the farnesyl pyrophosphate (FPP) synthase and lead to the same outcome [2].

Pharmacokinetically, they are very hydrophilic and poorly absorbed by the gastrointestinal tract due to their poor lipophilicity. Systemically available BPs disappear very rapidly from plasma and about 50% of the absorbed drug is taken up selectively by the skeleton. The rest is excreted in urine without being metabolised, as renal excretion is the only route of elimination [4]. The skeleton has a very high capacity to retain BPs, which, after bone uptake, are slowly released into the circulation and eliminated from the kidneys. The relative contribution of these two procedures to the overall obliteration from plasma differs significantly among BPs [5].

Nevertheless, long-term BP use is associated with multiple rare but severe adverse effects. More specifically, adverse effects include events of the gastrointestinal tract, such as abdominal pain, nausea, dyspepsia, vomiting and diarrhea [6], osteonecrosis of the jaw [7] and the maxillae [8], osteonecrosis of the external ear canal [9], hypocalcaemia [10, 11], atypical femoral fractures [12] or subtrochanteric fractures [13], carpal tunnel syndrome [14], renal failure [15], oesophageal cancer [16], uveitis with macular edema [17], interface dermatitis [18], orbital inflammation [19] and non-contact allergenic drug-induced baboon syndrome [20]. Recently, according to Cibickova et al. [21] experimental evidence has been provided that supports the idea of alendronate inhibiting cholesterol biosynthesis on the central nervous system. Therefore, awareness is being raised at the effects of BPs on the peripheral nervous system as well.

Reviewing of scientific bibliography revealed that there are not many research efforts regarding the influences of BP use either on the central or on the peripheral nervous system. In our report, we have studied the effects of BP use on the femoral nerve.

## Methods

### Ethical approval

This study was approved by the Bioethics Committee of the Medical School of the Aristotle University of Thessaloniki.

### Study design

Twenty female Wistar rats, 12-month old, weighing approximately 500 g, were used in the experiment. Rats were housed in stainless steel cages, with one rat per cage, 12 h light–dark cycle and relative humidity (35%) and temperature control (23 ± 2 °C).

The animals were randomly allocated into two groups: Group A, the research group, that consisted of 10 animals, and Group B, the control group, that also consisted of 10 animals. Alendronate (Fosamax, Merck) was administered per os to animals of Group A at a dose of 0.05 mg kg^−1^ body weight per week dissolved in 3 cc normal saline for a period of 13 weeks. The drug was administered thirty minutes prior to breakfast. The dose was calculated according to the usual human dose [22, 23]. The duration of the study was limited to 13 weeks and after euthanasia, the femoral nerve of the animals was removed and specimens were processed for electron microscopy examination. In this research we sampled three areas from each femoral nerve fiber (a total of 30 areas from each group), with every nerve part given the equal chance of being sampled.

### Transmission electron microscopy

Femoral nerve tissue samples were sectioned into < 1 cm^3^ pieces. They were placed into glutaraldehyde 3% for 2 h, followed by 1 h into osmium tetroxide (OsO_4_) 1%. Staining was performed with uranyl acetate 1% for 16 h and then samples were dehydrated with increased ethanol concentrations. Samples were embedded into Epon resin and ultra-thin sections (60–90 nm) were taken. Finally, sections were stained with Reynolds’s stain. Samples were observed under a TEM JEOL 1011 in 80 kV (Japan).

### G ratio measurements

Images were processed with National Institutes of Health ImageJ software [24] for quantification. The G ratios of myelinated fibers were calculated as the ratio of the axonal diameter to the diameter of the myelinated fiber as measured using ImageJ software (G ratio calculator plugin). Moreover myelin thickness was examined in four different locations and mean ± SD value was assessed [24].

### Statistical analysis

The results of study were analysed using IBM SPSS Statistics ver. 24. Continuous data were expressed as the mean difference (MD) and Standard Deviation (SD). The Kolmogorov–Smirnov test was used to determine whether our data were skewed or not. Furthermore t test for independent variables and both *p* ratio and confidence of interval (CI) were used to compare the data extracted. *p*-values of 0.05 and CI of 95% were used as thresholds for statistical significance.

## Results

### Morphological comparison

**Fig. 1:**
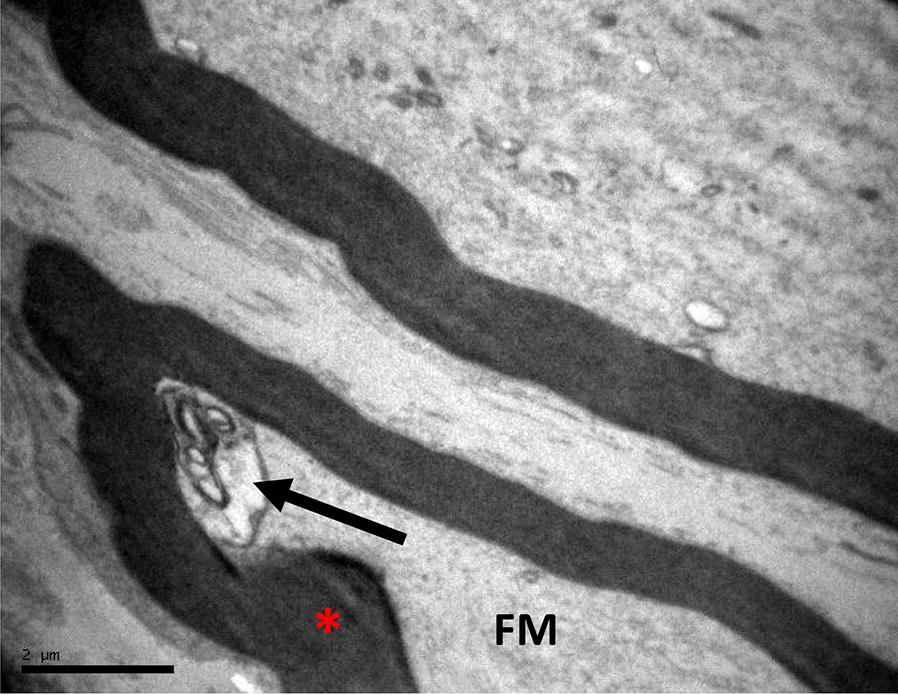
Control group: Femoral nerve (FM) with no major changes with the exception of some spots of thickness of the nerve myelin sheath (red asterisk) and small detachment of the axon (arrow)

In control group, most of the nerves were physiological and few of them showed detachment of the axon and small local thickening of myelin sheath (Fig. 1).

**Fig. 2:**
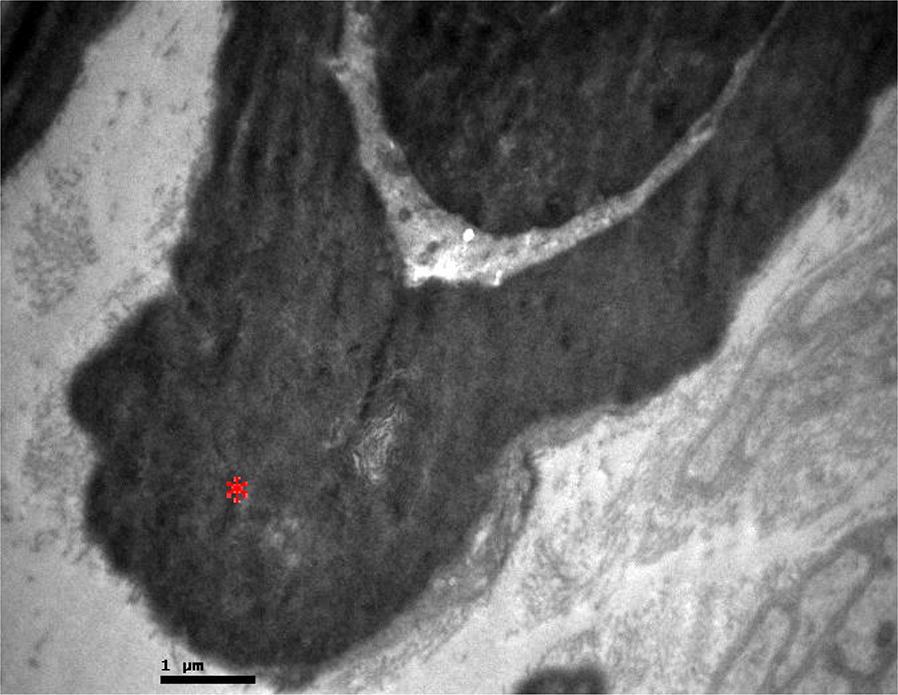
Research group: myelin thickening (red asterisk)

**Fig. 3:**
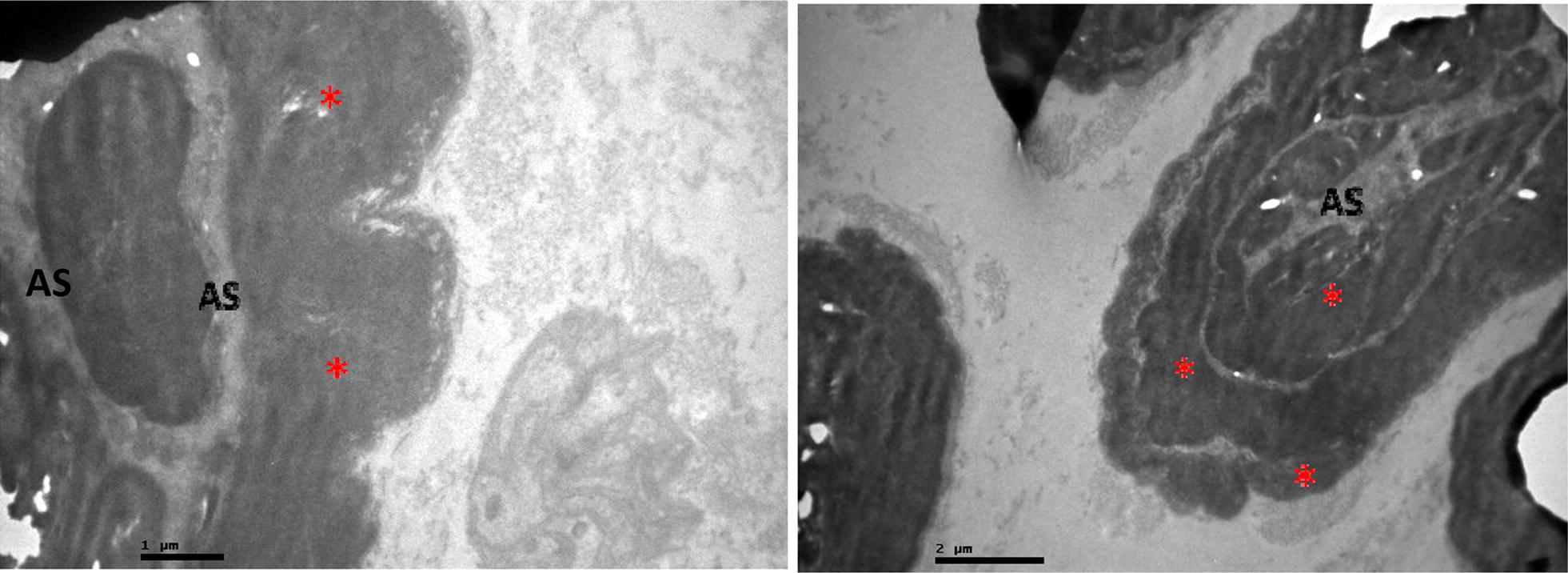
Effects of alendronate on femoral nerve of research group. Myelin thickening (red asterisk) and αxon strangulation (AS)

**Fig. 4:**
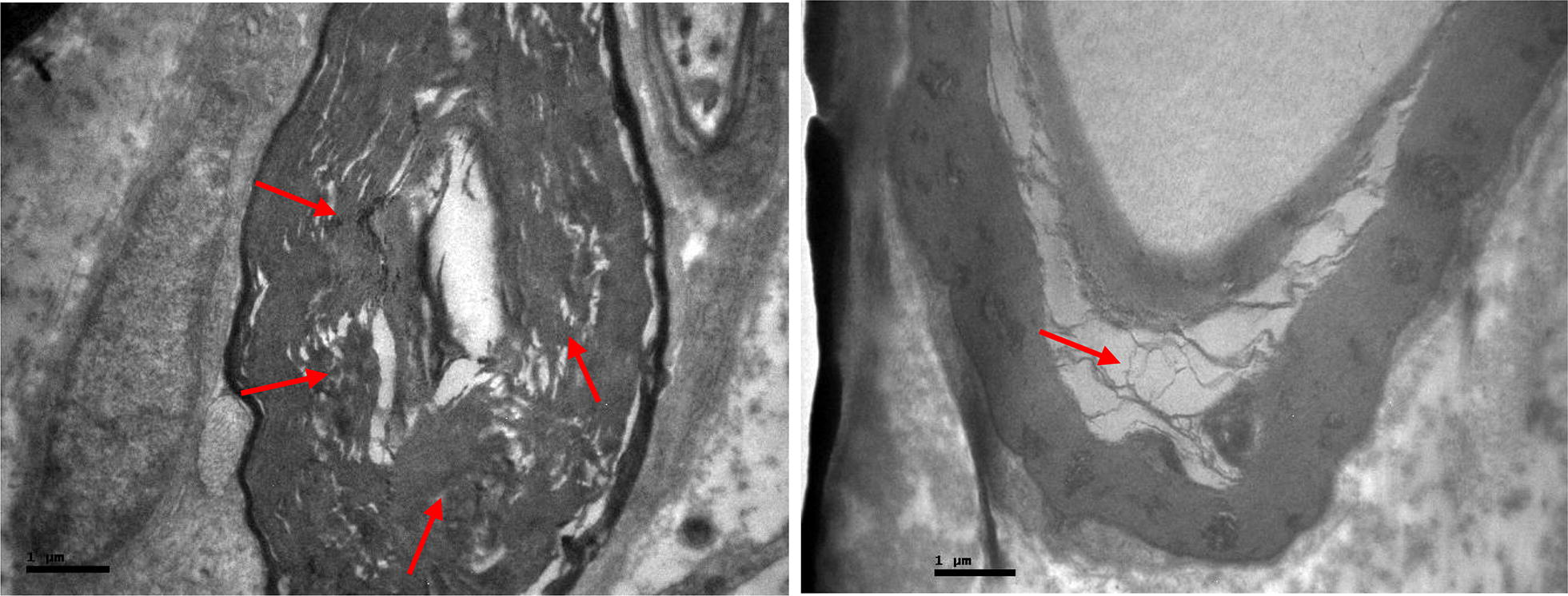
Effects of alendronate on femoral nerve of research group. Myelin partial disorganisation and vacuolization (red arrow)

In research group, in all samples the degenerative changes were more profound. These changes were the detachment of the axon, the thickening of the myelin sheath that varied from light to severe which lead in axon strangulation (Figs. 2 and 3) as well as the vacuolization and disorganization of the myelin sheath (Fig. 4).

### Statistical analysis

On top of these morphological changes a difference between the axon diameter of control and research group, myelin thickness and G ratio were found. The G ratio is defined as the ratio of the inner axonal diameter to the total outer diameter and has been utilised by several researchers. The G ratio may indicate abnormal reciprocal signaling between the axon and the myelinating Schwann cell, or may highlight thin myelin or conversely thin axons.

These measurements were recorded in different neuron sites of both groups. Τhe minimum, maximum, mean value and standard deviation were recorded. As seen in Table 1 there is a difference between research and control group. Furthermore, there was a statistically significant difference of the G ratio between the two groups [*p *< 0.05 and CI (95%): (− 0.19, − 0.011)] (Table 2). In Table 3, the results of the independent samples t test are presented.

**Table 1:** Morphological parameters of femoral nerve in research and control groups

	Research group	Control group
Axon like diameter	6.04 ± 1.50	17.80 ± 4.39
Myelin thickness	1.78 ± 0.48	4.09 ± 0.79

**Table 2:** Mean value of G ratio for femoral nerve

	Research group	Control group
G ratio	0.60 (std. error mean 0.03)	0.71 (std. error mean 0.02)

**Table 3:** Difference in G ratio for femoral nerve in rats of control and research groups

	t Test		95% CI of the difference	
	Mean difference	Std. error difference	Lower	Upper
G ratio				
Equal variances assumed	− 0.104	0.044	− 0.196	− 0.012

## Discussion

Rushton was the first researcher who evaluated the G ratio in Central Nervous System (CNS) and peripheral nerves. Since Rushton derived an optimal theoretical G ratio of 0.6 [25], many studies attempted to address the matter. According to Chomiak and Hu, a theoretically optimized G ratio both for central nervous fibers (0.77) and for peripheral nervous fibers (0.6) can be calculated [26]. Although the theoretical measurements produced by Chomiak and Hu algorithm fall into small range with the ones expected by the observed G ratio in the literature (G ratio observed = 0.76–0.81) in the CNS, there is a noticeable difference between his measurements and the G ratio in peripheral nerves.

More specifically, Bega et al. [27] used Wistar rats to study the G ratio of the femoral nerve and whether age and training can be related to changes in the nerve fibers. According to their analysis of structural changes of the femoral nerve, the mean axon diameter was 4.2 ± 0.2 μm in sedentary and 5.6 ± 0.3 μm in trained rats, the mean myelin sheath thickness was 2.4 ± 0.3 μm in sedentary and 1.8 ± 0.28 μm in trained rats and the mean G ratio 0.60 ± 0.12 in sedentary and 0.64 ± 0.11 in trained rats [27].

The values reported in literature for the morphologic measurements of the femoral nerve in Wistar rats are not complying with the ones we found in our study. The mean Axon like Diameter in our control group of Wistar rats was 17.8 ± 4.39 μm, the myelin thickness 4.09 ± 0.79 μm and G ratio 0.71 ± 0.86. On the other hand, there was a significant reduction of all three variables studied in the femoral nerve of the research group. Considering that G ratio is reliable for assessing axonal myelination and function of the femoral nerve, the use of alendronate in Wistar rats in dosage suggested for human patients can be considered a distinct factor affecting the normal function of femoral nerve fibers. On top of that, nerve conduction velocity (NVC), a reliable factor used for measuring the nerve’s condition, is closely related to a decrease of axon diameter and myelin of the myelinated fibers [28]. Although we did not measure the NVC in our experiment, it would be a very interesting variable, for our colleagues to use in such studies in the future. In any case, more research is needed to determine how these changes will affect the nerve’s function in long lasting treatment with BPs.

The difference between the measurements in our control group and the measurements we found in the bibliography could be due to the small sample we used. However, there are no large studies that have recorded these parameters of the femoral nerve in Wistar rats and that could be used as reference in our experimental study. To our knowledge no systematical bias could have caused this difference.

## Conclusions

In conclusion, the literature values for the morphologic measurements of the femoral nerve in Wistar rats are not complying with the ones we found in our study. There was a significant reduction of all three variables (the mean axon like diameter, the myelin thickness, G ratio) studied in the femoral nerve of the research group in contrast to control group. Our study demonstrates a possible correlation between alendronate administration and femoral nerve’s function, nevertheless due to the small specimen further research is needed.

## Data Availability

Please contact author for data requests.
